# Beyond Detection: Towards Actionable Sensing Research in Clinical Mental Healthcare

**DOI:** 10.1145/3699755

**Published:** 2024-11-21

**Authors:** DANIEL A. ADLER, YUEWEN YANG, THALIA VIRANDA, XUHAI XU, DAVID C. MOHR, ANNA R. VAN METER, JULIA C. TARTAGLIA, NICHOLAS C. JACOBSON, FEI WANG, DEBORAH ESTRIN, TANZEEM CHOUDHURY

**Affiliations:** Cornell Tech, Cornell University, USA; Cornell Tech, Cornell University, USA; Cornell Tech, Cornell University, USA; Columbia University, USA; Northwestern University Feinberg School of Medicine, USA; New York University Grossman School of Medicine, USA; Weill Cornell Medicine, USA; Geisel School of Medicine at Dartmouth College, USA; Weill Cornell Medicine, USA; Cornell Tech, Cornell University, USA; Cornell Tech, Cornell University, USA

**Keywords:** passive sensing, mHealth, behavioral health, mental health, clinical decision support, user-centered design, human-computer interaction, digital phenotyping, digital biomarkers, personal sensing, context-awareness

## Abstract

Researchers in ubiquitous computing have long promised that passive sensing will revolutionize mental health measurement by detecting individuals in a population experiencing a mental health disorder or specific symptoms. Recent work suggests that detection tools do not generalize well when trained and tested in more heterogeneous samples. In this work, we contribute a narrative review and findings from two studies with 41 mental health clinicians to understand these generalization challenges. Our findings motivate research on actionable sensing, as an alternative to detection research, studying how passive sensing can augment traditional mental health measures to support actions in clinical care. Specifically, we identify how passive sensing can support clinical actions by revealing patients’ presenting problems for treatment and identifying targets for behavior change and symptom reduction, but passive data requires additional contextual information to be appropriately interpreted and used in care. We conclude by suggesting research at the intersection of actionable sensing and mental healthcare, to align technical research in ubiquitous computing with clinical actions and needs.

## 1 Introduction

Ubiquitous computing technologies continue to permeate our everyday lives. Sensors embedded in these technologies collect fine-grained data on location, sound, light, and movement [15] that can approximate behavior and physiology [2, 27, 119]. Researchers have repurposed this data to monitor the health and well-being of individuals and populations by uncovering associations between digital data, clinical measurements, disease risk [41, 49, 102], and well-being [21]. From a clinical perspective, ubiquitous technologies are appealing because they gather *passive sensing data*: real-time data automatically collected from patients’ everyday lives with little-or-no user participation, enabling the collection of clinically-relevant information both inside and outside the clinic, revealing insights that are difficult to discern during infrequent, point-in-time clinical encounters [123].

Almost 15 years of interdisciplinary work in ubiquitous computing, psychology, and psychiatry has focused on using passive sensing data to train machine learning tools that detect mental health status [17, 31, 170]. In this work, we use the term **detection** to refer to research on machine learning models that process passive sensing data to detect individuals in a population experiencing a mental health disorder or specific symptoms. Passive sensing-detection research intersects with areas of computational psychiatry interested in the real-time quantification of mental health from passive data, called *digital phenotyping* [160], *digital biomarkers* [8], *personal sensing* [93, 114], or *behavioral sensing* [113]. Passive sensing data have been applied to detect mental health disorders including depression [6, 177], schizophrenia [18, 168], anxiety [78, 79], and bipolar disorder [16, 56]. The stated “value” of detection research is to create a low-burden method, using passive sensing, to continuously measure mental health outside of the clinic, and identify and manage emergent symptoms early-on [8, 77].

Despite this research and promise, recent work suggests that passive sensing-mental health detection tools do not generalize well when they are trained and tested in more heterogeneous samples. By *generalization*, we refer to the performance of task-specific (eg, depression prediction) detection models on data unseen during model training [131]. By *heterogeneity*, we focus on inter-individual differences, with data collected from similar points in time, devices, and data collection methods. We focus on generalization across individuals because it has been the focus of recent detection research [4, 105, 106, 178]. For example, Table 1 summarizes studies that have trained machine learning models with smartphone passive data to classify individuals experiencing clinically significant depression. These studies suggest that depression classification tools are more accurate in homogeneous samples, for example a sample of collocated undergraduates (AUC=0.81) [170], compared to heterogeneous samples (eg, a larger U.S.-wide sample, AUC=0.55) [4]. Studies using wearable sensing [129], detecting continuous depression symptoms [110, 150, 182], major psychiatric events in schizophrenia [3, 22, 124], and individuals living with bipolar disorder [57] have also struggled to find strong, generalizable associations between passive data and mental health. We recognize these studies are examples, and a systematic review is needed to conclude that detection models do not generalize well. Yet, these findings question the effectiveness of detection, and motivate alternative opportunities for passive sensing research in mental healthcare.

**Fig. 1.**
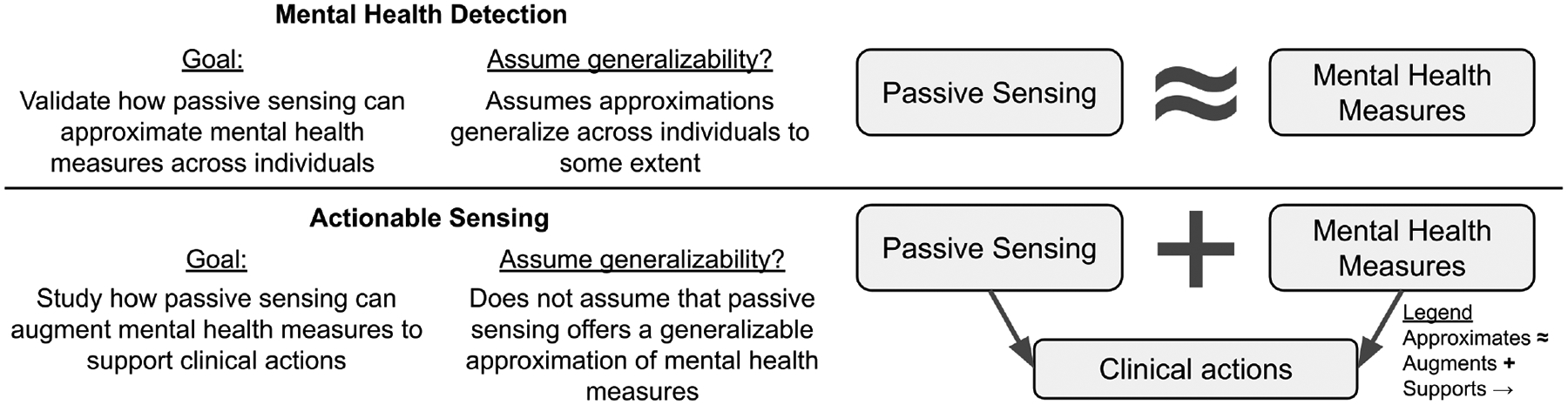
Contrasting the goals of mental health detection and actionable sensing research. Passive sensing refers to passively collected data on observable behavior and physiology, like sleep duration or heart rate variability. Mental health measures include measures of mental health disorders (eg, major depressive disorder) or specific symptoms (eg, loss of interest or pleasure), collected from patient self-reports or clinician-rated scales.

In contrast to detection, a growing line of work in ubiquitous computing and digital mental health has explored how passive sensing can complement traditional mental health measures (eg, patient symptom self-reports, clinician-rated scales) to support clinical actions and decision making [53, 123, 146]. In this study, we use the term **actionable sensing** to refer to research studying how passive sensing data can augment traditional mental health measures to support actions in clinical mental healthcare. Actionable sensing offers a contrasting and alternative research direction to detection work (Figure 1). Detection research focuses on validating how passive sensing can approximate traditional mental health measures, and assumes these approximations generalize across individuals to some extent. In contrast, actionable sensing research does not assume that passive sensing offers a generalizable approximation, and instead envisions passive and traditional mental health data working together to drive more effective care [123].

In this work, we explored through literature review and user studies with mental health experts both (1) detection generalization challenges, and (2) alternative opportunities for actionable sensing research in mental healthcare. We explored these two objectives through the following research questions:

**RQ1:** From literature, why do passive sensing-mental health detection tools not generalize well?
**RQ2:** How do mental health experts interpret these generalization challenges?
**RQ3:** What opportunities do mental health experts identify for actionable sensing research in clinical care?
**RQ4:** Based upon the opportunities identified from RQ3, how could passive sensing data impact care?

We explored RQ1 by conducting a narrative review of ubiquitous computing, psychiatry, and psychology literature (Narrative Review: Section 3). We then conducted a formative interview study with 21 mental health experts, specifically practicing clinicians, to answer RQ2 and RQ3 (Study 1: Sections 4 and 5). Finally, to answer RQ4, we conducted a design probe study where 20 additional mental health clinicians were shown collected passive sensing data, and we explored how this data could impact care (Study 2: Sections 6 and 7). Our contributions are:

- Findings from a narrative review and formative interviews explaining why generalizable detection is challenging: inter-individual differences in mental health symptom presentation and reporting result in patient-specific passive signals associated with symptom changes. These findings imply that generalization challenges will be difficult to solve with ubiquitous computing research because they stem from inherent reliability challenges in mental health measurement.
- Formative interview findings supporting alternative opportunities for actionable sensing research in clinical care. These opportunities focus on using individual-level passive sensing data to identify patient-specific presenting problems; generate insight on the relationships between observed behavior, physiology and symptoms; identify treatment targets that could be modified to reduce symptoms; and monitor patients’ treatment response.
- Findings from a second, design probe study that refine these opportunities for actionable sensing research. Our findings chart a path towards future research exploring the use of contextual information that illuminate individual-level, causal associations between passive sensing data and symptoms. These associations could support behavioral interventions and a more effective mental healthcare.

## 2 Related Work

We first provide an overview of the types of data relevant to this work (Section 2.1). We then give an overview of passive sensing-mental health detection research and perspectives on generalizability (Section 2.2). Finally, we conclude with a review of actionable sensing research in mental healthcare (Section 2.3).

### 2.1 Data in Mental Healthcare

There are a variety of data used in mental healthcare most relevant to this work. Traditional data for measuring mental health symptoms include validated *self-reports*, like the PHQ-9 [90] for depression, or the GAD-7 for anxiety [149]. These self-reports assess persistent, 2-week symptoms, while other self-reports can assess in-the-moment symptoms. For example, clinicians may ask patients to self-report their sleep quality each morning, a practice called *experience sampling* [152]. Self-reports, broadly, are considered *active sensing* data because they require users’ active participation for data collection [123]. Outside of active sensing, clinicians can assess symptoms by conducting clinical interviews [59] and quantify symptom severity on a clinician-rated symptom scale [173]. Clinician-rated symptom scales may be more frequently used for conditions, like schizophrenia, where impairment affects patients’ ability to self-report [133]. Scales and other data collected during clinical interviews are used to determine whether a patient meets criteria to be diagnosed with a mental health disorder (eg, major depressive disorder, schizoaffective disorder) [59, 90].

Clinicians may also wish to collect and review data on patients’ observed behavior and physiology in their everyday environments. These signals can be estimated using *passive sensing* data: data collected from digital devices with little-to-no user effort [123]. Passive sensing data can be collected through smartphones or wearables [17], online platforms, such as social media [140] or search engines [23]. Raw data from these devices and platforms may include GPS location data [137], phone usage, voice, light sensor, and accelerometer data [168], text message data [111], and the frequency and/or content of digital interactions [22]. Sleep patterns, like onset/wake times, can be estimated by tracking phone usage [2], or with physiological and behavioral signals collected by many consumer wearable devices [60]. Passive sensing offers a low-burden method to collect data related to mental health that is less arduous to administer than self-reports [123]. But, while clinical guidelines exist for using self-reports, like the PHQ-9, in care [145], similar guidelines do not currently exist for other types of active or passive sensing data, and their use tends to be case-by-case [123].

### 2.2 Passive Sensing-Mental Health Detection and Generalization

Researchers in ubiquitous computing have explored using passive sensing to detect different types of mental health outcomes. These studies build *detection models* using supervised machine learning. These models process passive sensing data to approximate a *ground truth* mental health measure, for example, a self-reported active sensing measure such as the PHQ-9 [97, 136, 170, 178], or in-the-moment symptoms [7, 33, 105]. Studies have also used clinician-rated symptom scales as detection outcomes [169]. Symptom severity scores can be thresholded to produce classification outcomes associated with mental health disorder diagnosis [90]. Researchers have also developed passive sensing tools to detect diagnostic status using outcomes from “gold-standard” structured clinical interviews as ground truths [59, 80].

The performance of detection models are measured using the *generalization accuracy*, estimating how well the model approximates a ground truth mental health measure within data not included in model training [138]. Researchers debate what level of generalization is necessary before introducing models into clinical settings. Studies show that clinical machine learning models are unlikely to generalize across heterogeneous samples [175], and it may be more practical to train and validate models in targeted populations [68, 148]. However, developing models for smaller populations can lead to bias, where models are underparameterized, do not capture the true data distribution [131], and overestimate performance [165]. In addition, there are no best practices to identify target populations with similar passive sensing-mental health relationships, and these relationships are heterogeneous within demographic and socioeconomic subgroups [4, 7]. In this work, it is not our intention to suggest what level of generalization is necessary, but instead explore what generalization challenges reveal about our ability to detect mental health with passive sensing.

### 2.3 Actionable Sensing Research

The term “actionable sensing” comes from sensing and AI research describing actions with data-driven tools [24–26, 98, 180]. Similar to actionable sensing, human-AI interaction researchers have studied how AI can enable actions in clinical care, for example, as an aid to clinical decision-making. These researchers have studied how AI outputs, often predicting disease risk, can be viewed alongside clinical data to support care [101, 147, 179]. In this work, we situate actionable sensing research analogously, to study how passive sensing can augment traditional mental health data to support actions in clinical care.

Researchers in ubiquitous computing, human-computer interaction, and digital mental health have explored opportunities for actionable sensing research in mental healthcare. For example, personal informatics researchers study how passive data can inform conversations between caregivers [36, 51, 53, 67]. Passive data can also support interventions, for example, by measuring the treatment effect in N-of-1 trials [44], or enabling just-in-time adaptive interventions (JITAIs) [89, 118, 130]. Interviews with mental health clinicians have described specific opportunities for passive data to enable actions in care including reflection and behavior change [46, 123, 146]. These findings align with principles of behavior therapy [37, 172] and the U.S. National Institute of Mental Health’s (NIMH) call for focused research on *treatment targets* – eg, disruptive behaviors – over “symptoms”, as targets are the “mechanism of action by which the intervention might ultimately modify…symptom(s) of interest” [76]. Contrasting detection research, actionable sensing does not assume that passive sensing data can approximate mental health across a population. Instead, actionable sensing research suggests that there are specific care contexts – for example, patient-specific behavior change in psychotherapy – where relationships between passive sensing and mental health exist and are useful to monitor [123].

Informed by these ideas, we conducted a narrative review followed by two studies with mental health experts, specifically practicing clinicians. We wished to first explore mental health-passive sensing detection challenges, and then offer alternative research directions on actionable sensing in mental healthcare.

## 3 Narrative Review (RQ1)

**Fig. 2.**
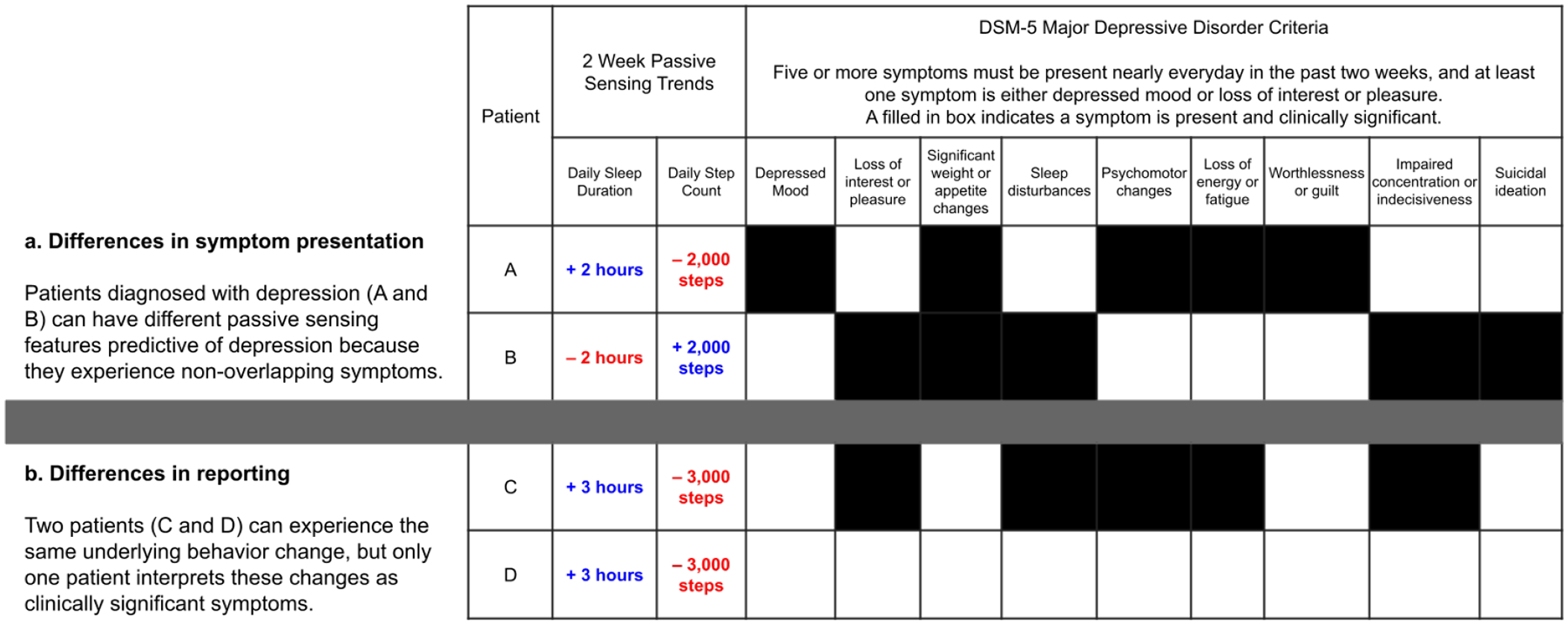
Why do passive sensing-mental health detection tools not generalize well when trained and tested in more heterogeneous samples? A visual description of our hypothesis from Section 3 suggesting that detection tools do not generalize well due to inter-individual differences in **a.** mental health symptom presentation and **b.** reporting. The table shows data for four patients, including 2-week passive sensing feature trends, as well as depression symptoms patients report experiencing (the black squares). Symptoms represent DSM-5 diagnostic criteria for major depressive disorder [9, 163].

Motivated by RQ1, we looked to generate a hypothesis suggesting why passive sensing-mental health detection tools do not generalize well. We conducted a narrative review, which surveys literature to support hypothesis generation [127]. Our narrative review suggested the following hypothesis, summarized in Figure 2:

**Hypothesis:** Passive sensing-mental health detection tools do not generalize well when trained and tested in more heterogeneous samples due to inter-individual differences in mental health symptom presentation and reporting.

To conduct the narrative review, we followed the process of literature accumulation and synthesis described in [127]. First, we accumulated and synthesized recent literature suggesting researchers have struggled to find generalizable passive features that detect mental health (Section 3.1). We then synthesized relevant literature in psychiatry and psychology to explore why these generalization challenges arise (Section 3.2).

### 3.1 Inter-individual Differences in Predictive Passive Sensing Features

#### 3.1.1 Personalization Improves Model Performance.

Detection models must assume that associations between passive sensing features and mental health symptoms generalize across individuals [131]. Literature suggests this is not the case, and that personalized machine learning models tend to perform better than models trained on an entire sample. Taylor et al. and Tseng et al. showed that models trained within specific groups improved symptom severity prediction [156, 162]. [105, 168] used a different personalization approach to improve performance by adding a small amount of data for each participant to models during training, and testing model performance on participants’ remaining data. Outside of mobile sensor data, personalization has improved mental health detection models trained with social media data [141].

#### 3.1.2 Predictive Passive Features are Heterogeneous.

Studies have also explicitly modeled individual and group (eg, demographic) differences in predictive passive features. For example, papers studying group-personalized models have found that passive features predictive of mental health differ across sample subgroups [156, 162]. [105] found that relationships between smartphone-sensed behavior and mood differ across individuals living within different countries, and that these differences impact detection model performance. Extending these ideas to a single-country sample, a recent study found that a depression detection model had variable performance across demographic and socioeconomic subgroups, and associations between passively-sensed behavior and depression differed across subgroups [4]. Other work has qualitatively compared individuals to identify differences across passive features predicting mental health. In [117], researchers found that wearable sensing measures of self-reported and physiological stress were highly heterogeneous across study participants. [3] conducted a similar analysis and identified that smartphone-sensed behaviors predictive of psychotic relapse were different across individuals.

#### 3.1.3 Challenges with Personalization.

Taken together, these studies suggest that (1) personalized models may improve detection and (2) inter-individual differences in predictive passive sensing features, amplified in more heterogeneous samples, may explain why models need to be personalized to improve detection accuracy. While these findings could imply that personalized models “are the answer” to improving detection tools, they also show that personalization is difficult in practice. As Nagaraj et al. state, future work should embrace “the individuality of stress; no two people experience stress exactly the same way” [117]. [178] describe that individual-level personalization is difficult because it requires fine tuning multiple machine learning models on small amounts of participant data. In addition, training and testing personalized models on small datasets can lead to bias [131, 165]. In the following section, we further explore: why are predictive passive features different across individuals? Are there inter-individual differences in mental health symptoms that explain these findings?

### 3.2 Inter-individual Differences in Symptom Presentation and Reporting

The prior section suggests that it is difficult to identify passive features that predict mental health reliably across individuals. We turned to literature in psychology and psychiatry to better understand why mental health detection with passive sensing is difficult.

#### 3.2.1 Inter-individual Symptom Differences.

First, we found evidence that individuals can be diagnosed with the same mental health disorder, but experience vastly different symptoms (*inter-individual symptom differences*). For example, Fried and Nesse found that among 3,703 individuals diagnosed with major depressive disorder, there were over 1,030 unique symptom profiles, and the most common profile was found in only 1.8% of the sample [66]. Network analyses of symptom profiles also show that symptoms are shared across disorders, and boundaries between disorders are unclear [28, 42]. These papers suggest that mental health disorders are ill-defined and are not a single construct, and Figure 2a describes why symptom differences can affect passive sensing mental health-detection tools. Two patients meeting criteria for major depressive disorder (A and B) can have different passive sensing features predictive of depression because they experience non-overlapping symptoms.

#### 3.2.2 Inter-individual Reporting Differences.

That said, differences across individuals discussed in Section 3.1 extend beyond disorder detection, and were also found in symptom detection (eg, mood, stress) models. Literature suggests this could be due to symptom *reporting differences*. For example, mental health questionnaires intentionally ask individuals to report persistent (eg, 2 week) symptoms, but studies find that some individuals are more likely to report recent or briefly elevated symptoms [11, 74]. For psychotic disorders, like schizophrenia, self-reports are also affected by *patients’ insight* – their awareness of symptoms — and self-reported symptoms change as patients gain insight [20]. Similar associations between insight and symptom severity have also been identified for individuals diagnosed with depressive disorders [181]. This work may explain why mental health self-reports are only intended for within-patient monitoring and screening [47, 62, 65]: self-reporting behaviors are likely more consistent within-patients, self-reports often overestimate disease prevalence [95, 158], and thresholds to distinguish clinically significant symptoms from continuous symptom scores are unclear [65]. Figure 2b shows why reporting differences can affect passive sensing-mental health detection tools. Two patients (C and D) can experience the same underlying behavior change, but only one patient interprets these changes as symptoms.

#### 3.2.3 Are Challenges Due to Self-report?

These findings could suggest that detection challenges are caused by patients’ self-reporting behaviors, and clinician-rated scales offer a potentially more reliable detection outcome. It is difficult to prove that clinician-rated assessments are more reliable than self-reports. Field trails of diagnostic criteria show that among 23 mental health disorders, the test-retest and inter-rater reliability (agreement by two clinicians diagnosing independently across clinical visits) was rated as questionable or unacceptable for 9 disorders, potentially because many of these disorders were highly comorbid and difficult to distinguish [40, 132]. Furthermore, studies show that clinicians often miss patient deterioration [72]. Researchers contest that clinician-rated and self-reported scales are synonymous, suggesting self-reports may be more predictive of treatment response and both types of scales complement each other in clinical care [43, 164]. This is why comprehensive neuropsychological evaluations, often used for diagnosis, are a multi-step process that draw from symptom scales, cognitive tests, structured interviews, observation, and collateral information from family and friends [32, 104], though full evaluations are impractical in large-scale, longitudinal detection research.

### 3.3 Narrative Review Conclusion

Taken together, this review hypothesizes that (1) mental health detection tools do not generalize well because passive features predictive of mental health vary across individuals, and (2) this variation may be explained by inter-individual differences in symptom presentation and reporting. This hypothesis suggests that mental health measurement challenges, which affect the assessments used as ground truths to train detection models, make it difficult to identify reliable associations between passive sensing data and mental health symptoms. Solving these challenges may be beyond the scope of ubiquitous computing research in the absence of better ground truths. In the rest of this work, we present findings from user studies with mental health experts that further explore these challenges. In addition, we looked to suggest alternative opportunities for actionable sensing research that are not predicated upon generalizable associations between passive sensing data and mental health measures.

## 4 Study 1 Methods: Formative Interviews

We conducted interviews with mental health experts to interpret the detection model challenges hypothesized in the literature review (RQ2) and identify alternative opportunities for actionable sensing research (RQ3). We recruited mental health clinicians as participants to clarify our reading of the clinical literature with experts who observe the heterogeneity of mental illness in their practice. In addition, we wished to identify with our participants specific clinical needs that could be supported with actionable sensing.

Methodologically, we drew from work in user-centered design [84, 122, 123] to explore mental health clinicians’ current use of data – defined broadly (see Section 2.1) – and how passive data, specifically, may be used within future models of care. In this section, we detail the formative study procedures, including participant recruitment (Section 4.1), and how data was collected and analyzed (Section 4.2). All study procedures were approved by the coauthors’ institutional review boards (IRBs).

### 4.1 Participant Recruitment

We enrolled as participants mental health clinicians, including psychiatrists, clinical psychologists, licensed clinical social workers (LCSWs), and licensed mental health counselors (LMHCs). We intentionally recruited providers from these different clinical orientations to better understand how data is used in multiple aspects of treatment, from medication management, managed by psychiatrists, to psychotherapy, often delivered by clinical psychologists and social workers [108]. Participants were recruited via a combination of convenience, purposive, and snowball sampling [52, 70]. Specifically, the first author sent a recruitment email and flier to staff working in psychiatry departments (where multiple types of mental health clinicians practice) across the United States. Interviewed participants often forwarded the recruitment message to their colleagues or to trade organization that have mental health clinicians as their members. Potential participants were asked to provide informed consent after being provided information about the study procedures.

### 4.2 Data Collection and Analysis

Interviews were held via Zoom over two 1-hour sessions, attended by the first three authors, and participants were reimbursed $30 per hour for their time. In the first session, participants described how they used data as a part of their current clinical practice. In the second session, participants described how data could be used within future models of care. Specific interview questions were broad to allow for on-the-spot adaption and probing [19], and included: (in session 1) *what types of data do you use to inform your clinical practice? are there certain types of patients you use this data with? how does this data inform care?*; (in session 2) *what data would you use in the future to inform care outcomes? to improve care quality?*

Interviews were recorded with participants’ permission, transcribed by a professional service, and de-identified by the first author. Transcripts were analyzed using a reflexive thematic analysis approach adopted from [29], combining both inductive and deductive elements: codes and themes arose from the data, but were guided by our specific research questions and literature synthesized in the narrative review [30]. The first author qualitatively coded transcripts to develop an initial codebook. Codes were refined, a final codebook was developed, and all transcripts were recoded using the final codebook. Themes were developed from the codes by the first author. The second and third authors, who participated in the interviews, validated that the themes and quotes accurately represented participants’ views.

## 5 Study 1 Findings: Formative Interviews

Interviews occurred from October 2023 through January 2024. 21 mental health clinicians participated in the formative interviews (see Table 2). Out of these participants, 19 participants completed the full 2-hour study. One participant (C27) completed only 1 hour of the study, and another participant (C36) was only available for a 30 minute interview. Findings were derived using data from all 21 participants. Due to the small sample size and to protect anonymity, we chose to not collect or present more specific demographic information, including intersectional identities, and we did not interpret how participants’ identities influenced our findings. Participants are quoted using study IDs (eg, C45) to retain anonymity.

### 5.1 Clarifying Why Inter-individual Differences Make Detection Difficult (RQ2)

#### 5.1.1 Inter-individual Differences in Symptom Presentation.

Participants supported that symptom presentation within conditions differ across individuals and specific sections of the population. For example, C32 highlighted how there are “more specialized depression scales for older adults, or more specialized depression scales for postpartum women because there’s just so many symptoms.” Some participants preferred to use detailed scales with more specific symptoms because symptom nuances were important for treatment. One participant, C25, mentioned that “the PHQ [a short depression self-report] is usually a screener and it won’t account for things like delusions, guilt or taking care of yourself, and it’s only nine questions, but the HAM-D [a clinician-rated depression scale] is a lot more nuanced, so I wouldn’t use it to screen a patient, but I would use it when I’m monitoring someone’s symptoms.” C23 agreed, stating “you do have to know a little bit more than just the number. You actually need to see what the answers are on individual [symptom] questions.”

Participants also mentioned how patients with different symptoms may present with different problematic behaviors in treatment. As one participant stated, treating depression for some patients might look like “I’d be able to get out of bed and get to work on time”, but for other patients it may look like “I’d be much more present with my children” (C37). This is important for detection because it implies that behaviors associated with symptom change, even for the same disorder, differ across patients (Figure 2a). Another participant believed that, in some cases, it made more sense to focus on behaviors over symptoms in treatment, stating how they start monitoring patients “with symptom measures, assuming that was the reason that someone came in. And then I look at their goals and goal progress. Does that line up with the symptoms or do those things seem to be discrepant in some way? Because some goals may be that ‘I want to get along better with my partner’ and that’s not linked with depression or anxiety as closely as other things might be” (C43).

#### 5.1.2 Inter-individual Differences in Symptom Reporting.

Participants also explained that inter-individual symptom reporting differences could be explained by recall bias, when patients have difficulty recollecting symptoms, or choose to not report specific symptoms. For example, though validated symptom measures, like the PHQ-9 and GAD-7, ask patients to recall persistent symptoms, one participant noted how patients often report transient symptoms, because “some people are not insightful enough to think about, ‘how have I felt on average over the last two weeks?’ You catch a 12-year-old on a bad day and they’re going to answer the assessment [with all high scores] and make you think that you need to send them to an intensive program” (C38). Another participant noted how the context surrounding scale administration can affect recall, stating that “sometimes the patient does not feel comfortable fully disclosing their answers. We will see a discrepancy sometimes between how they fill out questionnaires with their doctor and how they fill it out with a mental health professional” (C37). One participant gave an example of a patient “who did not endorse any suicidality to the research assistant but did with me in our session together, and the measure was the same” (C39).

Given these concerns, we asked participants how reporting differences would influence our ability to interpret symptom scores across patients and identify generalizable passive features. C43 believed that “people are reporting as accurately as possible, but their understanding of what these measures mean in their everyday life and how these numbers map onto their experience shifts over time.” Another participant explained how “every patient fills in their questionnaires differently, but it’s relative to how they usually function in life” (C42). If reported symptoms are relative to baseline functioning, individuals can experience similar life changes, and only some individuals will experience and interpret these changes as symptoms, explaining why passive feature changes map to mental health symptoms in some individuals, but not others (Figure 2b). A participant agreed, stating that “what’s a 10 to me [on a scale] might not be a 10 to you”, but symptom scores are still useful because they are “data driven in nature, trackable, and are a jumping off point for more conversations” (C38).

### 5.2 Identifying Alternative Opportunities for Actionable Sensing Research (RQ3)

**Fig. 3.**
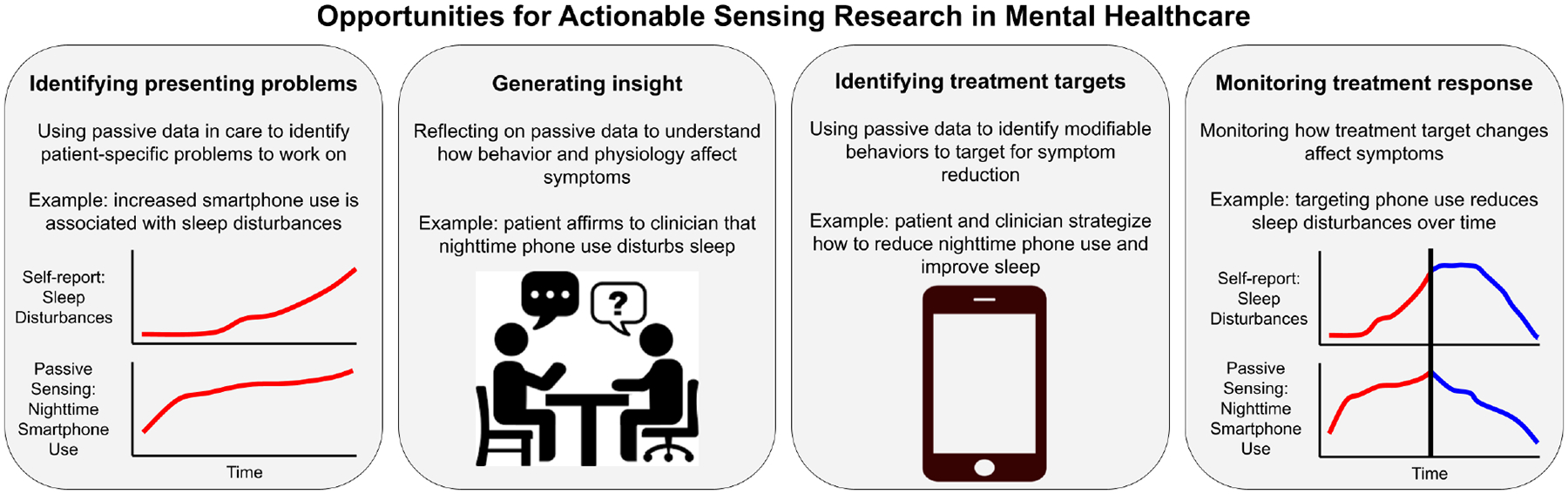
Opportunities for actionable sensing research in mental healthcare, identified in Section 5.2.

In addition to clarifying our hypothesis, we wished to identify with participants alternative opportunities for actionable sensing research (RQ3). These opportunities are summarized in Figure 3 and described in the following sections.

#### 5.2.1 Identifying Presenting Problems.

Participants described how data highlighted patient-specific presenting problems. For example if a patient self-reported “symptoms, let’s say sleep disturbance” the data would “inform a deeper assessment what’s going on at night” and help clinicians better understand “how does the patient prepare for bed? What’s getting in the way?” (C37). Participants believed that passive sensing data could help them more efficiently and effectively identify presenting problems. One participant mentioned how “traditionally you just deal with whatever a person is bringing in” and “try to do a detailed inquiry, not just take everything at face value, but really probe”, and passive sensing could “bring evidence, tracking the patient in their daily life” (C37). Another participant described how passive data could inspire questions to further understand behavior, specifically “is my patient getting up 20 times a night because they have an enlarged prostate and need to pee every five seconds, or because they’re super anxious?” (C24).

#### 5.2.2 Generating Insight.

Participants described how data helped patients generate insight: a greater understanding of themselves and their symptoms. One participant mentioned, in the context of patients with suicidal ideation, that gaining insight, specifically realizing the “connection between negative emotions and suicidality” was a first step towards managing symptoms (C39). This participant was interested in how passive data could improve insight by shedding light on how behaviors impact symptoms, mentioning that patients often “think they’re sleeping better than they probably are” and “the objective [passive] data could suggest that your body and brain are not actually resting as much as they can” or “challenge the distorted view that I have poor sleep” (C39). Another participant noted how patients “have difficulty with objectively monitoring their own experiences” and thus saw “for things like substance use, sleep, behavioral stuff that we can monitor easily, I see a lot of utility in that [passive sensing]” (C34).

#### 5.2.3 Identifying Treatment Targets.

Participants described how they used data to manage patients’ specific presenting problems by identifying treatment targets – modifiable behavioral or physiological signals that mediate downstream mental health symptoms. One participant, a child and adolescent psychologist, described that getting their patients to “school is a target” as well as “their ability to engage in exposures” (C30), which were everyday, fearful, situations that their patients, who were living with obsessive-compulsive disorder (OCD), typically avoided. Our participants were interested in how passive data could be used to identify and monitor patient-specific treatment targets. For example, one participant stated that for bipolar disorder, where “the main treatment that you do is social rhythm therapy” where patients aim to “get to sleep at a similar time” and you “actually have them track what time they go to sleep” and “literal sleep data would be amazing for that” (C42). Another participant gave a specific example on how passive sensing data from a wrist-worn actigraphy sensor informed treatment. The participant was treating “a seven year old who was insistent that she was staying in bed all night, but her mom said she was tired all the time.” The participant gave the patient an actigraph, and upon reviewing the data, found that the patient “was awake and playing with the dogs”, and that she asked the patient’s mom “to take the dogs out of the room, and two weeks later, the patient was sleeping fine” (C35).

#### 5.2.4 Monitoring Treatment Response.

Participants also used data to understand patients’ specific treatment response, including what treatments patients respond to, and how patients respond to treatment. As C43 described, data gave “more insight into how they respond, giving me a sense of like, is treatment working? Are we on the right track? Or are we off track?” Participants highlighted the ways in which passive data could give them tangible, longitudinal data to show patients’ treatment response. For example, C23 mentioned how “people who are depressed are usually less energetic, they’re less motivated. So if you see people are walking more, or moving more, that’s something else that you can think about” or “if the patient tells me that she’s been walking twice as much as usual and that her mood is better, then I’ll take it. That’s good news” (C31).

### 5.3 Study 1 Conclusion

Our formative interview findings highlight both the complexities of detection while inspiring alternative opportunities for actionable sensing research. These opportunities focus on how patients and their clinicians could identify passive sensing data relevant for care and act on this data to reduce symptoms. In the rest of this paper, we describe findings from a second study that refine these opportunities and inform future research on passive sensing in clinical mental healthcare.

## 6 Study 2 Methods: Design Probe (RQ4)

We conducted a follow-up study to explore how the specific opportunities for actionable sensing research identified in study 1 could impact care (RQ4). Specifically, we conducted a mixed-methods design probe study, where we showed mental health clinicians real, de-identified passive sensing and self-reported mental health data, and asked them qualitative and quantitative questions to explore using passive data in care. In this section, we detail our study methodology. Section 6.1 details the visualization tool we created to display passive and self-reported data. We then detail the study design in Section 6.2, and participant recruitment, data, and analysis procedures in Section 6.3. Study procedures were approved by the coauthors’ IRB.

### 6.1 Visualization Tool

**Fig. 4.**
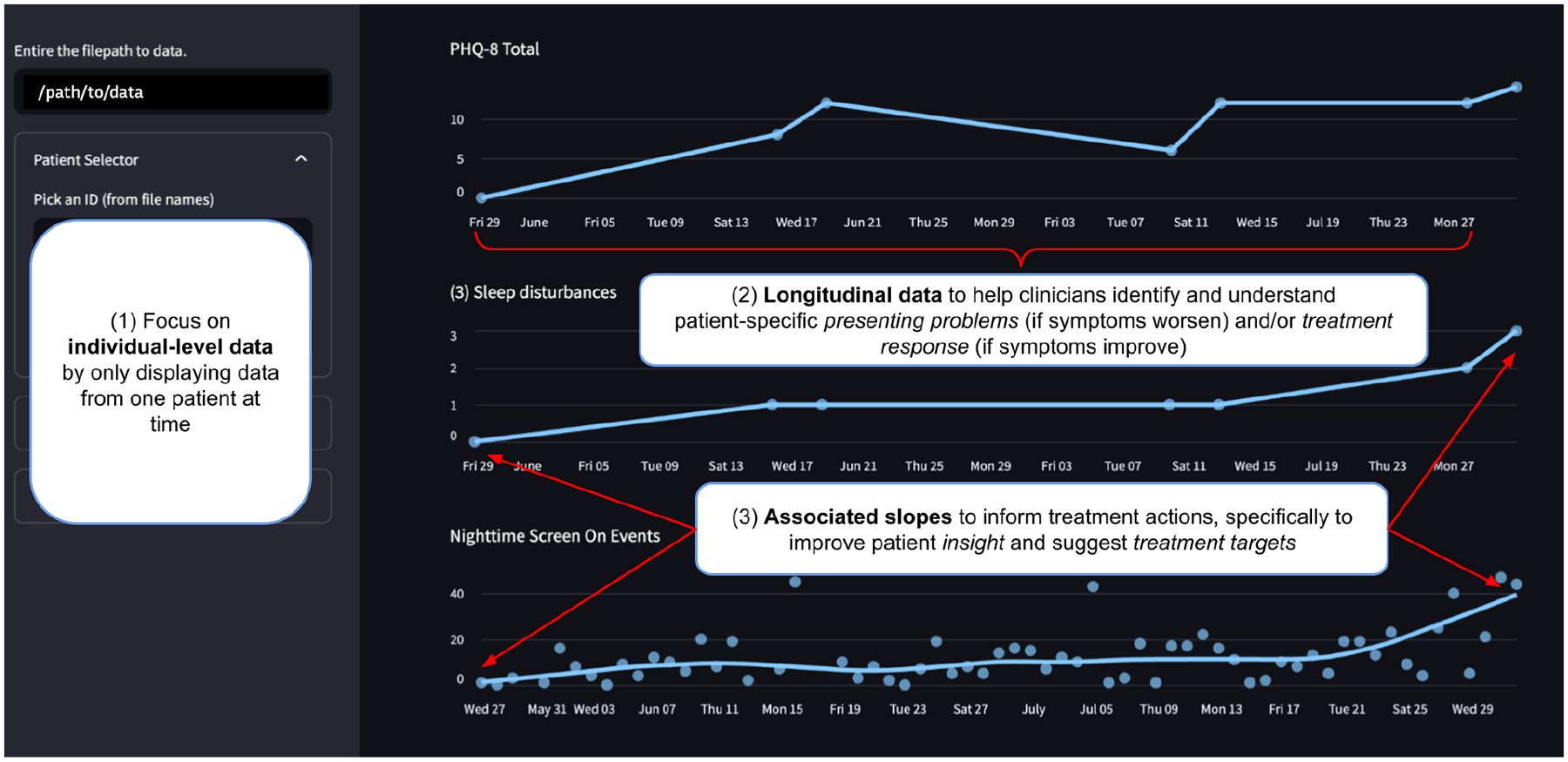
The design probe. Labeled affordances are further described in Section 6.1, and are motivated by our findings in Section 5.2. Dots represent data points, and lines connect data points in the PHQ-8 Total and symptom (sleep disturbance) graphs, but indicate average trends (a LOESS line) for passive data, in this case, “Nighttime Screen On Events”.

We built a design probe using the Streamlit Python library [153]. Our study goal *was not* to validate the utility of this specific probe, or to identify broadly generalizable conclusions about the use of passive data in care. Instead, in the design probe tradition [5, 69, 166], we wished to deeply study the impact of using passive data to support clinical actions. The de-identified data the tool displayed was collected during a U.S.-based NIMH-funded study to identify associations between smartphone passive sensing data and depression symptoms (see [4, 110, 150] for data collection methods). A screenshot of the tool can be found in Figure 4. Note that we use the word “patient” to refer to individuals whose data was displayed on the design probe. The probe had the following affordances:

1. **Individual-level data**: Findings in Section 5 suggest that clinicians primarily use data at an individual-level. We thus displayed data on the tool for one patient at a time.
2. **Longitudinal data**: Findings in Sections 5.2 suggest that clinicians were interested in using passive data to identify patient-specific presenting problems and treatment response. We displayed longitudinal total depression symptom severity scores, self-reported using the PHQ-8 [92], responses to specific PHQ-8 questions as symptoms, and passive sensing data to suggest presenting problems (if symptoms worsen) or treatment response (if symptoms improve). *PHQ-8 Total* scores range from 0 to 24 and higher scores indicate greater symptom severity. Symptom scores range from 0 to 3, where higher scores represent more frequent symptoms. We displayed two passive sensing-symptom pairs. 1) The first PHQ-8 question, measuring patients’ *(1) Loss of Interest/Pleasure*, which was paired with passive sensing data measuring the daily *Percentage Time at Home*: the percentage of time a patient spent at home throughout a day. Increased time at home can suggest social isolation, a sign of loss of interest or pleasure [171], and improving real-life social contact can reduce depression symptoms [109]. 2) The third question on the PHQ-8 measuring *(3) Sleep Disturbances* paired with daily *Nighttime Screen On Events*: screen on events recorded between 12–6 AM. Screen on events can indicate sleep disturbances [54, 134], and reducing late night phone use can reduce sleep disturbances [100].
3. **Associated slopes**: Findings in Section 5.2 suggest how clinicians perceive using passive data to improve patient insight by identifying changes in behavior and physiology associated with mental health symptoms. We thus displayed data suggesting associated slopes between mental health self-reports and passively sensed-behaviors. In addition, our formative findings describe how passive data could be used to identify and monitor treatment targets (reduce phone usage to decrease sleep disturbances; reduce time at home to decrease loss of interest or pleasure). We hoped the data would inspire conversations to explore associations in data as treatment targets.

### 6.2 Study 2 Design

**Fig. 5.**
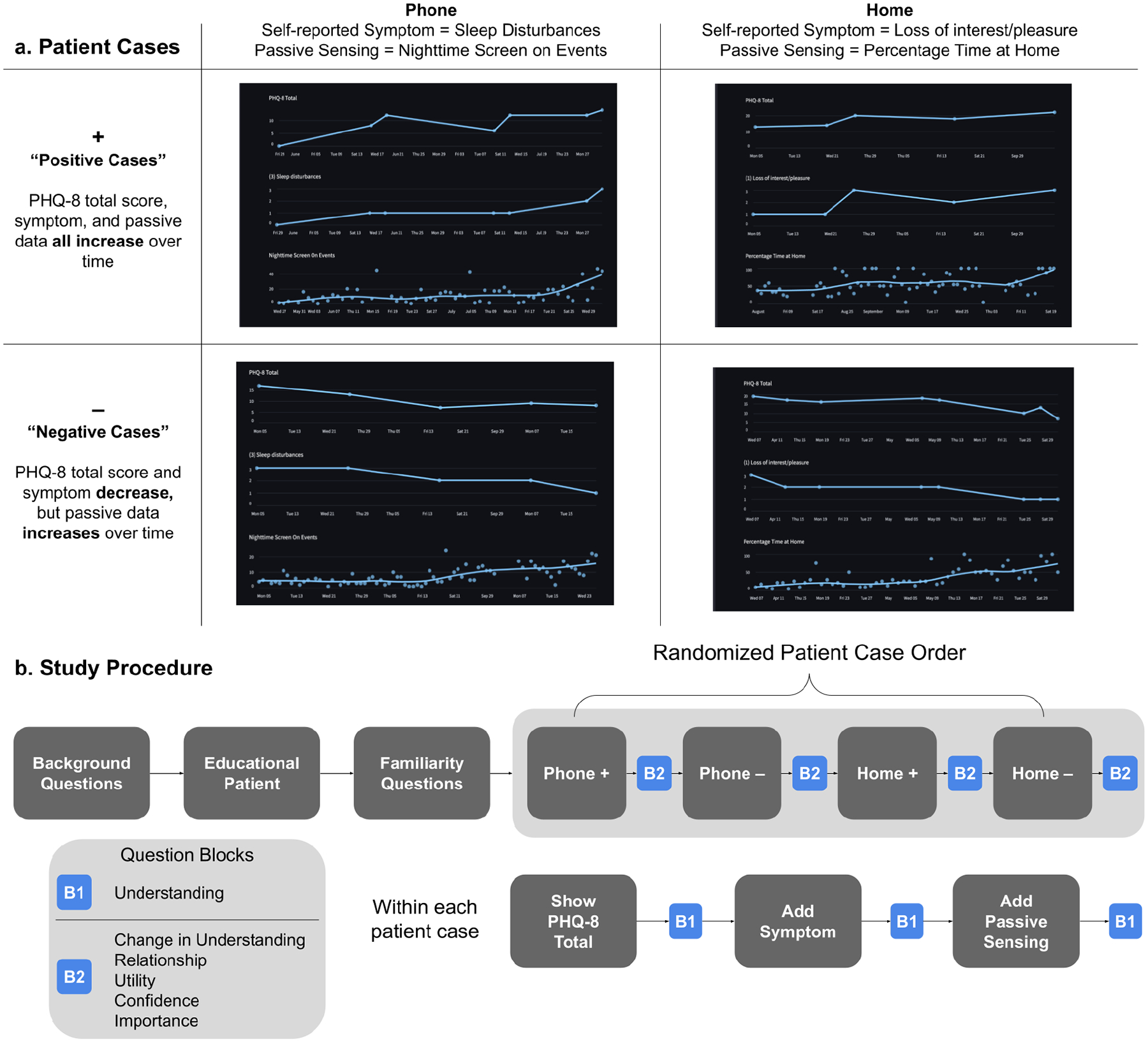
Study 2 design. **a** describes the four patient cases participants viewed in the study. **b.** describes the study procedure. We gradually showed participants data (the PHQ-8 total, symptom, passive sensing) within each case, and intermixed question blocks to understand how different data types impacted participants’ understanding of patients, and interest in using passive data to inform treatment actions. See Section 6.2 for more details.

Figure 5 summarizes the study design. One participant attended each study session, and we conducted the study over Zoom. After consenting, participants provided information about their clinical background. We then introduced participants to the design probe, showing data from an “educational patient” to teach participants about the different data types the probe displayed. After learning about the data, participants rated their baseline familiarity with each data type: *from your prior experience, how familiar are you with each type of data (PHQ-8 total, symptom, passive sensing) we have reviewed? 1 (no familiarity) to 5 (lots of familiarity)*.

#### 6.2.1 Patient Cases.

We then showed participants data from four different patients (*patient cases*), one patient at a time. The order of the patient cases was randomized across participants to reduce order bias. During each case, participants were first shown a patient’s longitudinal PHQ-8 total score. We then added symptom (sleep disturbances OR loss of interest/pleasure) data to the screen, followed by passive data (nighttime phone use OR percentage of time at home). Similar to [147], for each patient, we kept the data type presentation order fixed, adding data types one at a time so participants could gradually acquaint themselves to the patient case.

The four patient cases were inspired by prior work investigating AI clinical decision support tools [101, 147]. In this work, clinicians were shown AI-generated recommendations that were both in concordance with and subverted their expectations. Analogously, we showed participants two patient cases that contained expected behavior-depression relationships from literature [110, 139, 159] (**positive, + cases**), and two cases that subverted these expectations (**negative, – cases**) to understand how passive data impacts care when it confirms and challenges assumptions about how behavior impacts mental health. Four patient cases with apparent and sound associations were chosen from collected data in collaboration with a non-participant practicing clinician who co-authored this work. The cases (described in Figure 5a) were:

**Phone+** PHQ-8 total score, sleep disturbances, and nighttime screen on events *all increase*.
**Phone−** PHQ-8 total score and sleep disturbances *decrease*, but nighttime screen on events *increases*.
**Home+** PHQ-8 total score, loss of interest/pleasure, and percentage time at home *all increase*.
**Home−** PHQ-8 total score and loss of interest/pleasure *decrease*, but percentage time at home *increases*.

### 6.2.2 Question Blocks.

We asked participants quantitative and qualitative questions as we showed them patient data. These questions were motivated by concepts including working alliance, specifically how well clinicians understand patients’ treatment goals [71, 99], technology acceptance [38, 45, 161] and perceived usefulness [101, 147], which may influence clinicians’ use of passive data. Within each patient case, as we added a data type to the screen (eg, the PHQ-8 total score, the symptom, and then passive sensing data), we asked participants *what observations could you make about this patient, using this data?*; *how you would approach treating this patient, once they came in for their visit, using this data?*; and quantitatively to rate *on a scale of 1 (low) to 5 (high), based upon the data on the screen, how much do you feel you understand this patient?* This question block is labeled as **B1** in Figure 5b, and we called this quantitative question the **Understanding** question in Figure 5 and Section 7.

After we finished showing participants data for a single patient, we asked the following quantitative questions (B2 in Figure 5b): **Change in Understanding**, *did the smartphone [passive sensing] data change your understanding of this patient? 1 (little-to-no change) to 5 (great change)*; **Relationship**, *did you see a relationship between the smartphone data and the patient’s self-reported symptoms? (no relationship; as the smartphone data increased, the symptom increased; as the smartphone data increased, the symptom decreased)*; **Utility**, *how useful was the smartphone data to help you understand this patient? 1 (not useful) to 5 (very useful)*; **Confidence**, *if a patient shared this smartphone data with you, how confident would you feel using this data as a part of treatment? 1 (not confident) to 5 (very confident)*; and **Importance**, *rank order the importance of each type of data (PHQ-8 total, symptom, and smartphone) for understanding and treating this patient. (1 = highest rank, 3 = lowest rank)*. We probed participants to collect qualitative data that further explained numerical responses.

### 6.3 Recruitment, Data Collection, and Analysis

We recruited and reimbursed mental health clinicians as participants using the same methods described in Section 4.1. Recruited participants did not participate in study 1. The first two authors attended the study sessions. Interviews were recorded with participants’ consent, transcribed, and de-identified by the first author. The first two authors analyzed the transcripts following the same reflexive coding procedure described in Section 4.2.

## 7 Study 2 Findings: Design Probe (RQ4)

20 mental health clinicians completed study 2 from January through March 2024 (see Table 3). We refrained from conducting significance tests of the quantitative results because the small sample size (n=20) would result in low statistical power and undermine any conclusions [34]. Instead, we contribute a descriptive analysis of our findings. Participants are quoted using study IDs (eg, U10) to retain anonymity.

**Fig. 6.**
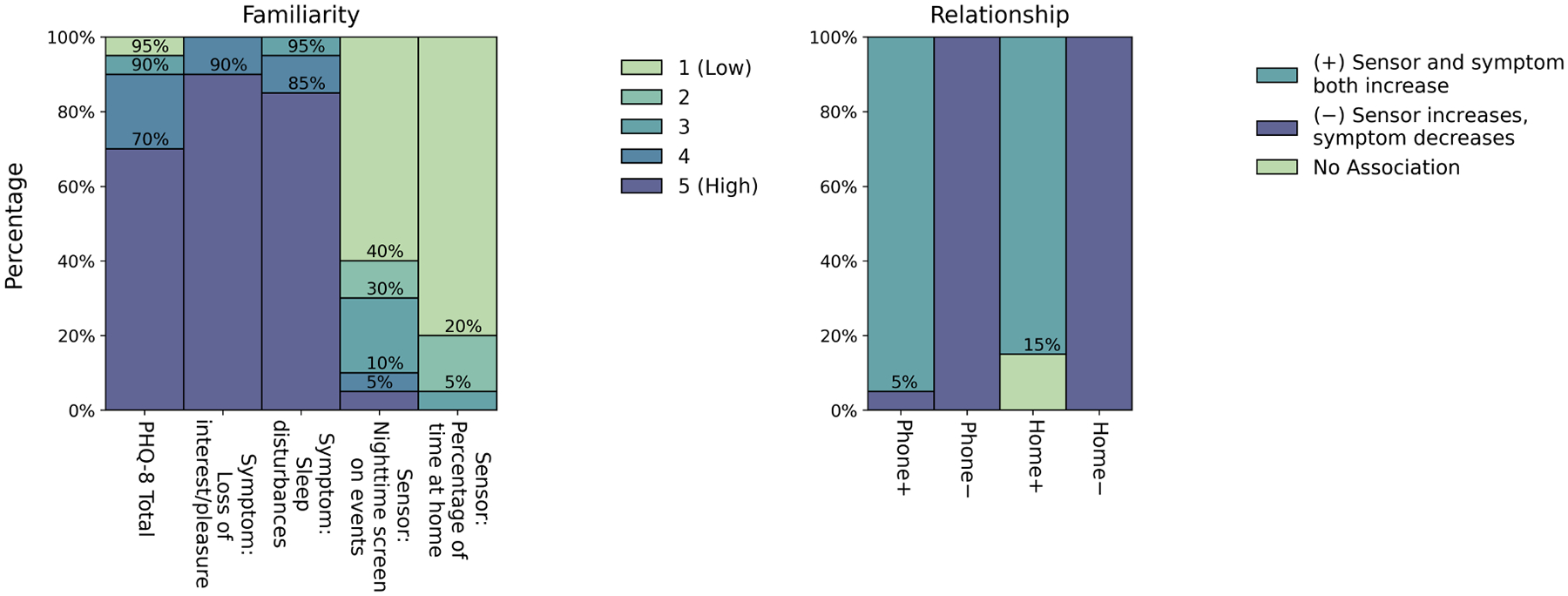
Baseline familiarity and validating the relationships in the data. **Left.** Histograms show response distributions to the question: *from your prior experience, how familiar are you with each type of data we have reviewed? 1 (no familiarity) to 5 (lots of familiarity)*. The x-axis describes the data types participants were shown. **Right**. We validated if participants noticed the intended relationships between the passive and mental health symptom data slopes within each patient case. Bars are specific to the patient cases described in Figure 5, and the + and − on the x-axis ticks identifies the intended associations. On all histograms, numbers above the bars indicate the cumulative distribution (eg, 90% of values ≥ 3).

The majority of participants reported a high baseline familiarity (Figure 6, left) with the PHQ-8 total score (1 = no familiarity, 5 = high familiarity, n=18, 90% responses ≥ 4), loss of interest/pleasure (n=20, 100% ≥ 4), and sleep disturbance (n=19, 95% ≥ 4) symptoms, but low familiarity with the nighttime screen on events (n=14, 70% ≤ 2) and percentage of time at home (n=19, 95% ≤ 2) passive sensor data. Participants often described that while they had never used passive data in clinical practice, they often asked about “phone use, especially at night, especially if there’s sleep disturbances” (U21) and that they had experiences using and tracking “how much time patients spend on their phone at nighttime as part of sleep hygiene” (U14). In contrast, participants were slightly less familiar with the percentage of time at home measure, but participants could see the relationship between time at home, “isolating and things like that” (U07) and that “if you’re depressed, you might be spending a lot of time at home” (U13). Most participants were also able to identify the intended associations (Figure 6, right) between the passive sensor and mental health symptom data slopes for each patient case (identification rates: Phone+ n=19, 95%; Phone− n=20, 100%; Home+ n=17, 85%; Home− n=20, 100%).

### 7.1 Refining Opportunities for Actionable Sensing

#### 7.1.1 Identifying Presenting Problems.

**Fig. 7.**
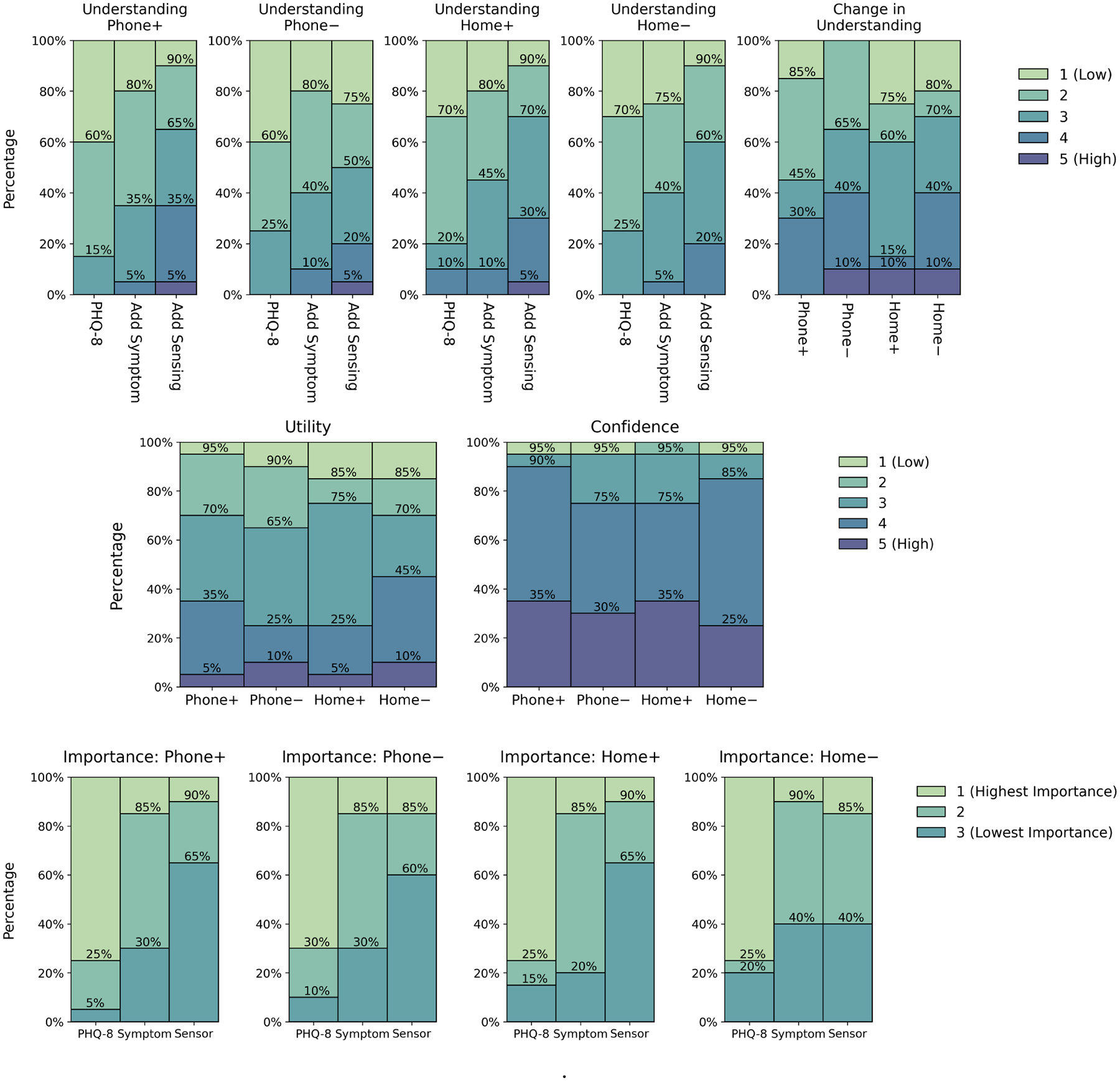
Study 2 quantitative response distributions. **Top, Left**. *On a scale of 1 (low) to 5 (high), based upon the data on the screen, how much do you feel you understand this patient?* Each histogram is specific to the patient cases described in Figure 5. Participants were always first shown patients’ PHQ-8 total scores, then symptom, and finally smartphone sensing (x-axes). **Top, Right**. *Did the smartphone data change your understanding of this patient? 1 (little-to-no change) to 5 (great change)*. **Middle, left**. *How useful was the smartphone data to help you understand this patient? 1 (not useful) to 5 (very useful)*. **Middle, right**. *if a patient shared this smartphone data with you, how confident would you feel using this data as a part of treatment? 1 (not confident) to 5 (very confident)*. Each bar is specific to the patient cases on the x-axis described in Figure 5. **Bottom**. *Rank order the importance of each type of data for understanding and treating this patient*. Each histogram is specific to the patient cases described in Figure 5. The x-axis describes the data types shown to participants. On all histograms, numbers above the bars indicate the cumulative distribution (eg, 90% of values ≥ 3)

Participants felt the passive data increased their understanding of the patient when it more clearly identified meaningful signal on patients’ presenting problems. For example, 12 (60%) participants stated that the nighttime phone use data increased their understanding (score change ≥ 1, see Figure 7, top-left) when phone usage increased with sleep disturbances over time (Phone+ case). Participants stated that the Phone+ case was in “line with what one might expect” and that they were “starting to have a more coherent view [of the patient], starting to develop some more clear hypotheses of what might be happening” (U08). Another participant mentioned how the passive data gave “validity to what’s actually happening with that patient” (U12). Participants, in general, were fairly confident using passive sensing data when they could more easily reason through the data to identify presenting problems. For example, 18 (90%) participants were reasonably confident (score ≥ 4, on a scale of 1, low confidence, to 5, very confident, Figure 7, middle-right) using the passive data in the Phone+ case because “there’s a clearer correlation with sleep” (U01).

#### 7.1.2 Challenging Assumptions.

Participants were also interested in passive data when it challenged their assumptions about how behavior affects mental health. For example, 9 (45%) participants ranked that the passive data led to a greater change in understanding when it subverted their expectations (higher score for − versus + in both Phone and Home cases, see Figure 7, top-right). In the Phone– case, where nighttime phone use increased but symptoms decreased, U07 stated that the data gave them “a different perspective”, and another participant interpreted that the data could indicate that the patient “works a night shift, maybe they’re asleep from 9:00 AM to 4:00 PM, so then they’re on their phone at night” (U06).

#### 7.1.3 Insight Generation.

Participants were interested in reflecting on the data with their patients to improve insight. For example, U16 mentioned how in the Home– case they thought that the patient could be “anxious and depressed, and if they stay home, the anxiety drops out and they feel a little bit better” and they could ask their patient “at sweet spots in your life do you like being at home?” to understand if time at home was “an avoidance strategy”. Another participant (U02) mentioned how time at home data could help patients gain insight because “the biggest issue you have with anyone who’s struggling with anxiety or depression is that they view everything through what they’re feeling in that moment” and “they’re not always the most accurate reporters”.

#### 7.1.4 Identifying Treatment Targets.

Participants stated how the passive data in the – cases may indicate treatment targets: behaviors that lead to symptom reduction. U20 mentioned how the Phone– case, where phone use increased but symptoms decreased, the patient could “wake up in the night, and they have to put on their hypnosis or meditation app”. U03 mentioned in the Home– cases that the passive data “is giving me some context to now we [the patient] spend more time at home and we feel better.” In contrast, some participants (7, 35%) participants believed that the nighttime phone usage data was more useful (higher utility score, Figure 7, middle-left) in the Phone+ case than the Phone– case, because in this case, they felt the treatment target was clearer. As one participant stated, in the Phone+ case they could explore with patients “strategies to minimize the use of the phone overnight and improve sleep” (U08).

### 7.2 Uncovering Challenges

#### 7.2.1 The Need for Context.

Many participants believed that the data would not be useful without additional contextual information to clarify associations between passive sensing and symptoms. Otherwise, their interpretation of the data “would all just kind of be shots in the dark” (U06). As U10 stated, “they couldn’t just use that data on its own” and “they would have to follow up” with the patient. U22 mentioned how in the Home+ case, that they did not “want to just assume that if you’re leaving the home you’re healthy and if you’re home, you’re not healthy”, stating that patients “could be avoiding something at home”. These quotes suggest that, in general, participants were more confident using the passive data to inspire follow up questions for patients, and not use the passive data, alone, to inform treatment actions. As U14 stated “numbers can mean anything”, and only felt confident using the data “if they [the patient] were sharing it and I would have the opportunity for context.” These findings explain why participants rarely ranked the passive sensing as the most important data type (Figure 7, bottom). As U15 stated that they “don’t think the passive data could be a sole intervention or treatment”, and U05 believed that “it was the combined data that enhanced the smartphone data value”. U14, agreed, stating that: “I would want to understand what their baseline for sleep is, to understand if it’s actually a sleep disturbance. So if they are an executive and they’re used to going on five hours of sleep, and now, they’re getting eight hours of sleep, for them, that might feel like they’re sleeping too much, but that might actually be healthy.”

The need for context was prominent when participants perceived passive sensing-symptom relationships as unexpected or ambiguous. For example, only 6 (30%) participants reported an increased understanding of the patient (score change ≥ 1, Figure 7, top-left) when nighttime phone use increased, but sleep disturbances decreased (Phone– case). In fact, 2 (5%) participants lowered their understanding scores for this case. U05 mentioned how they were “surprised that sleep has gotten better, but there’s more time on the phone”. Similarly, participants were split on whether the time at home data, in both cases, increased their understanding (Home+ n=10, 50%; Home– n=9, 45%). U22 found the implications of the time at home data ambiguous, and wanted “to look more at the research” to understand “are people who spend more time at home more depressed or less depressed?”

#### 7.2.2 Reckoning with Personal Beliefs.

Some participants reported that data types changed their understanding only when the associations confirmed their personal beliefs on how behavior affects mental health. For example, 6 (30%) participants in the − case, and 8 (40%) participants in the + case ranked that nighttime phone use led to a greater change (higher score, Figure 7, top-right) in understanding than the time at home data. U18 explained, the phone usage data”tells me more information” and “I wouldn’t expect someone to use a phone that much during the night” while they doubted that less time at home should improve mental health, stating that “I’m much happier at home”. Comparatively, only 5 (25%) participants in both +/− cases reported a higher change in understanding for the time at home compared to the phone use cases. This included U16, who stated: “for me, I find that if I don’t leave the house even during a single day, it’s a very different kind of day than when I go out and walk around the block”.

## 8 Discussion

Taken together, our findings present actionable sensing as an alternative research direction to detection while surfacing both opportunities and challenges for using passive sensing to support actions in clinical care. Specifically, our findings suggest that actionable sensing research can bring individual-level passive measures of behavior and physiology into care that highlight patients’ presenting problems, treatment response, and motivate inquiry on how behaviors and treatment impact mental health. That said, our findings also suggest that clinicians share different beliefs on how behavior impacts mental health, and require additional contextual information to interpret and integrate passive data in care. In this discussion, we consider these findings with the literature to clarify the future development of actionable sensing technologies for clinical mental healthcare.

### 8.1 Mining Contextualized, Longitudinal Multimodal Passive and Clinical Data

Our findings support designing technologies that help clinicians and their patients better navigate contextualized, longitudinal multimodal passive and mental health data. These technologies can assist with identifying presenting problems, treatment response, and allow clinicians and their patients to reflect on data to increase insight. These ideas intersect with personal informatics research [39, 51], recent work studying clinician-patient preferences for visualizing passive and self-reported data [36, 143], and “context-aware” computing applications [48].

One open research question is how to best identify relevant passive and clinical data highlighting presenting problems or treatment response, and integrate insights from this data into clinical workflows. Otherwise, clinicians may experience “information overload”, which could perpetuate burnout [135], and choose to not use passive data in treatment [123]. Many papers have used simple statistical approaches to identify passive sensing features associated with symptom changes [3, 105] that may be relevant for treatment, but our findings suggest that it would be difficult to validate if these associations are meaningful without additional contextual information. For example, recent work has explored how to create “context-aware” interventions that combine passive sensing and treatment data with patients’ specific goals and preferences [121]. Contextualized goals and preferences could be summarized with clinical and passive data by interactive, intelligent agents using AI [50, 120], and integrated into visualizations to guide clinicians and patients towards data relevant for care. Outside of building technology, passive data use in clinical care requires workforce and compensation changes, for example, hiring “digital navigators” who assist with data use [174], and developing reimbursement mechanisms for time spent reviewing passive data [154].

Another challenge is how to operationalize data sharing preferences. Patients can have varied preferences about sharing personal data with clinicians [115], and clinicians may be wary to receive and view passive data if they perceive data usage as a violation of patients’ privacy [123]. From a technical perspective, this calls for developing flexible methods of “tiered passive data access”, analogous to tiered access in research data sharing [13, 83], that allow patients and clinicians to seamlessly move from less (eg, percentage of time at home) to more (eg, fine-grained location) sensitive passive data as sharing preferences evolve in treatment. From a sociotechnical perspective, theories such as contextual integrity [125, 126] motivate creating norms and policies surrounding when and how passive data could be shared within clinical contexts.

### 8.2 Identifying and Monitoring Treatment Targets

In addition to supporting patient-clinician reflection, our findings support actionable sensing research on identifying and monitoring treatment targets. The concept of a “treatment target” persists across different types of behavioral therapy. For example, in behavioral activation, patients undergo a therapeutic process of focused behavior change to improve mood [73, 81]; in exposure therapy, patients expose themselves to fearful stimuli in order to reduce their fear [61]; in social rhythm therapy, patients regularize their routines to reduce sleep-wake cycle dysregulation and improve functioning [63]. In these therapies, the “target” of treatment is engagement in a specific task (behavior change, exposures, or routine regulation), and task engagement over the course of treatment reduces mental health symptoms [61, 157].

One question for actionable sensing research is: can passive sensing be used as a target to monitor task engagement? Recent work has found that clinicians are interested in using passive data to monitor engagement in psychotherapy [53]. But, using passive sensing as a treatment target assumes causality, for example, that decreased phone use should reduce insomnia. Establishing causality is challenging, and studies that identify individual-level, causal associations, specifically with small data, must reduce the risk of bias in their results. For example, [117] used a causal discovery algorithm to identify individual-level wearable sensor indicators causally associated with mental health. This work established static causal indicators, while passive data is longitudinal and causal associations may change over time [8]. The dynamic nature of passive data calls for adaptable, time series causal discovery methods, for example from [14, 85] for treatment target identification, as well as causal approaches to predict the effects of modifying treatment targets on mental health [58].

A prospective method for treatment target identification would be to modulate passive sensing variables by having patients take specific actions during treatment (eg, decrease nighttime phone), and tracking the effects of these actions. In this setting, treatment delivery must be randomized to establish causality, for example, borrowing methods from micro-randomized trials [88], or approaches specifically designed for small data, like N-of-1 trials [44]. Researchers have already studied mental health applications that create “micro-interventions” using passive sensing to track engagement in behavior change [64, 107]. However, a critique of this direction is that it reduces mental health to modifiable behaviors trackable through passive data [151], and our participants noticed the flaws in this assumption. For example, nighttime phone use may not cause poor sleep: a person may not sleep because they live in a loud urban environment. We intend for future actionable sensing research to develop technologies and design interactions that account for these external factors [128], as well as other social factors that influence device ownership, passive data collection, and capabilities for behavior change [167].

### 8.3 Monitoring Treatment Response to Improve Care Quality

Section 8.1 focused on methods to identify treatment response in the context of individual patient care. From a health systems and public health perspective, there are other motivations to identify indicators of treatment response. Global healthcare costs have increased, and governments are testing programs that hold health systems accountable for delivering treatments that improve patient outcomes and reduce cost [103, 176]. In mental health, there is no consensus for what measures should assess treatment outcomes and the quality of care delivery [55]. Existing mental health quality measures focus on process, not outcomes, for example, measuring how often clinicians screen for symptoms [86]. These quality measures may not reflect the complexities of mental illness in everyday life [12, 87], are often distal from patients’ and clinicians’ care needs [155], and are difficult to translate into concrete actions that improve patient care [35]. In addition, our findings and the literature show that mental health providers are inclined to rely on assessments and treatments informed by their clinical judgement over evidence and research [82, 96].

This creates opportunities in actionable sensing to study how passive data change throughout the course of treatment, and how practitioners can use this information to improve care quality. Studies have linked passive measures of activity with chronic disease and mortality [94, 102, 142], and have also identified associations between passive data and symptom reduction [1]. Yet, our findings motivate research into how passive sensing can measure treatment outcomes beyond symptom reduction. For example, treatment engagement is a proximal outcome of psychotherapy, and studies show that passive sensing can measure engagement in treatment [10, 53, 144]. Researchers have also used AI to identify effective patient-clinician interactions in psychotherapy [112], and insights can be fed back to clinicians to improve the quality of patient encounters [75]. We envision a world where passive sensing data promotes evidence-informed interventions, and insights from passive data are shared with clinicians and health officials to improve mental health services.

### 8.4 Limitations and Future Work

Our findings reflect our interpretation of the literature joined with the perspectives of 41 mental health clinicians, and should not be interpreted to reflect mental health clinicians as a whole. All participants in this study were based in the United States, and these findings are biased towards mental healthcare in the U.S. In addition, our design probe methodology allowed for in-depth engagement with shown data, but these probes did not explore all possible passive sensing-mental health relationships. We chose to conduct a narrative review instead of a scoping or systematic reviews, which give a more complete summary and/or synthesis of the literature. A more rigorous review may reach different conclusions. We interviewed mental health experts, specifically practicing clinicians, to understand how passive sensing can align with existing data practices and clinical needs. We plan to include patients’ perspectives, exploring use cases in both self-management and clinical care as future work. Finally, we hope that the research directions outlined in this discussion motivate exciting future work for ubiquitous computing researchers at the intersection of actionable sensing and clinical mental healthcare.

### 8.5 Conclusion

In this work, we call for more focused research on actionable sensing as a vision to bridge promising technical research in passive sensing with clinical actions and needs in mental healthcare. We present actionable sensing as an alternative to but not a replacement for detection research, and we hope that research in mental health detection using passive sensing data improves screening and assessment. Simultaneously, actionable sensing research can develop and evaluate technologies that bring passive sensing data on behavior and physiology into clinical encounters and improve care. We are excited to engage and collaborate with the community on designing innovative technologies that support the needs of patients, clinicians, and health systems, improve treatment outcomes, and mental health service delivery.

### 8.6 Positionality

The first, second, and third authors are graduate students in computer and information science. These authors recruited participants, collected, and analyzed all of the data. Two authors are both clinical researchers and practicing mental health clinicians who worked with the first author on the study protocols, including creating the design probe. All other authors were either clinical researchers, or researchers in computing and information science who contributed to the final manuscript. All authors were based in the United States.

## Figures and Tables

**Table 1.** Example performance differences across studies using smartphone passive sensing to detect clinically significant depression.

	Study	N	Sample Description	Depression Criteria	Performance
Lower heterogeneity	[139]	18	Individuals living across the United States	PHQ-9 ≥ 5	BA = 0.87
	[116]	57	Students at a university in the northeastern United States	ICD-10 criteria for major depressive disorder	AUC = 0.82
	[170]	83	Students at a university in the northeastern United States	PHQ-4 ≥ 3	AUC = 0.81
Higher heterogeneity	[178]	534	Students at two universities in the United States	PHQ-4 ≥ 3	AUC = 0.58 BA = 0.55
	[4]	650	Individuals living across the United States	PHQ-8 ≥ 10	AUC = 0.55
	[105]	678	Students from universities across 8 different countries	Positive versus negative mood score	AUC = 0.52
	[116]	5,262	Individuals living across the United States	ICD-10 criteria for major depressive disorder	AUC = 0.57

Heterogeneity refers to the number of participants (“N”) in each study and/or the diversity of participants in the study sample (see “Sample Description”). PHQ-x are validated self-reported depression symptom measures [90–92]. The reported performance for all studies except for [170] were computed by using cross-validation partitioning data by participants. [116] explored depression detection models in both small (N=57) and large (N=5,262) samples. [105, 178] focused on generalization across new datasets. [178] describes the validation accuracy from the model trained using three out of four collected datasets, validated on the fourth dataset. [105] describes the validation accuracy of the model trained and validated on all available data. AUC = the area under the received operating curve; BA = the balanced accuracy.

**Table 2.** Background information of formative interview study participants. Participants could list multiple practice settings. IQR = interquartile range

Study 1 participants	N=21 mental health clinicians
Median (IQR) years of clinical experience	9 (5 – 15)
Clinical training	11 Clinical Psychology 5 Psychiatry 4 Clinical Social Work 1 Mental Health Counseling
Practice setting	16 Academic Medical Center 8 Private Practice 2 Community Mental Health Center
Geographic location (in the USA)	18 Northeast 2 Southeast 1 West Coast

**Table 3.** Background information of mental health clinicians participating in study 2. These participants did not take part in study 1. Participants could list multiple practice settings. IQR = interquartile range

Study 2 participants	N=20 mental health clinicians
Median (IQR) years of clinical experience	16.5 (10 – 23.5)
Clinical training	7 Clinical Psychology 7 Clinical Social Work 6 Psychiatry
Practice setting	17 Private Practice 16 Academic Medical Center 14 Community Mental Health Center 10 Non-academic Health System
Geographic location (in the USA)	16 Northeast 2 Midwest 2 Southeast

## CCS Concepts:

• **Human-centered computing** → **Empirical studies in ubiquitous and mobile computing; Empirical studies in HCI**; • **Computing methodologies** → *Machine learning*; • **Applied computing** → Health informatics.
